# Vaccine-Derived Poliovirus Outbreaks and Events — Three Provinces, Democratic Republic of the Congo, 2017

**DOI:** 10.15585/mmwr.mm6710a4

**Published:** 2018-03-16

**Authors:** Mary M. Alleman, Rohit Chitale, Cara C. Burns, Jane Iber, Naomi Dybdahl-Sissoko, Qi Chen, Djo-Roy Van Koko, Raimi Ewetola, Yogolelo Riziki, Hugo Kavunga-Membo, Cheikh Dah, Rija Andriamihantanirina

**Affiliations:** ^1^Global Immunization Division, Center for Global Health, CDC; ^2^Division of Viral Diseases, National Center for Immunization and Respiratory Diseases, CDC; ^3^CDC, Democratic Republic of the Congo, Kinshasa; ^4^Institut National de Recherche Biomédicale, Ministry of Public Health, Democratic Republic of the Congo, Kinshasa; ^5^Immunization and Vaccine Development, World Health Organization, Democratic Republic of the Congo Country Office, Kinshasa; ^6^Immunization Unit, United Nations Children’s Fund, Democratic Republic of the Congo Country Office, Kinshasa.

The last confirmed wild poliovirus (WPV) case in Democratic Republic of the Congo (DRC) had paralysis onset in December 2011 (*1*). DRC has had cases of vaccine-derived polioviruses (VDPVs) documented since 2004 (Table 1) (*1*–*6*). After an outbreak of 30 circulating VDPV type 2 (cVDPV2) cases during 2011–2012, only five VDPV2 cases were reported during 2013–2016 (Table 1) (*1*–*6*). VDPVs can emerge from oral poliovirus vaccine (OPV types 1, 2, or 3; Sabin) polioviruses that have genetically mutated resulting in reversion to neurovirulence. This process occurs during extensive person-to-person transmission in populations with low immunity or after extended replication in the intestines of immune-deficient persons following vaccination (*1*–*6*). During 2017 (as of March 8, 2018), 25 VDPV cases were reported in three provinces in DRC: in Tanganyika province, an emergence with one VDPV2 case (pending final classification) in Kabalo health zone and an emergence with one ambiguous VDPV type 1 (aVDPV1) case in Ankoro health zone; in Maniema province, an emergence with two cVDPV2 cases; and in Haut Lomami province, an emergence with 20 cVDPV2 cases that originated in Haut Lomami province and later spread to Tanganyika province (hereafter referred to as the Haut Lomami outbreak area) and an emergence with one aVDPV type 2 (aVDPV2) case in Lwamba health zone (Table 1) (Figure) (*6*). Outbreak response supplementary immunization activities (SIAs) were conducted during June–December 2017 (Table 2) (*6*). Because of limitations in surveillance and suboptimal SIA quality and geographic scope, cVDPV2 circulation is likely continuing in 2018, requiring additional SIAs. DRC health officials and Global Polio Eradication Initiative (GPEI) partners are increasing human and financial resources to improve all aspects of outbreak response.

**TABLE 1:** Number of acute flaccid paralysis (AFP) cases with any vaccine-derived poliovirus (VDPV) in stool samples, by year of paralysis onset and province — Democratic Republic of the Congo, 2004–2017*^,†^

Province	Year												
	2004	2005	2007	2008	2009	2010	2011	2012	2014	2015	2016	2017	Total
Bas Uele	—^§^	—	—	—	—	1	—	—	—	—	—	—	1
Equateur	—	—	—	—	—	1	—	—	—	—	—	—	1
Haut Katanga	—	—	—	—	—	—	—	—	—	1	—	—	1
Haut Lomami	—	7	—	16	2	—	13	17	—	—	—	8	63
Kasai	—	—	—	—	2	3	—	—	—	—	—	—	5
Maindombe	—	2	—	—	1	—	—	—	—	1	—	—	4
Maniema	1	—	—	1	—	10	—	—	—	—	—	2	14
Mongala	—	—	—	—	1	2	—	—	—	—	1	—	4
Sud Kivu	—	—	1	—	—	—	—		—	—	—	—	1
Tanganyika	—	—	—	1	1	—	—	—	1	—	—	15	18
Tshopo	—	—	1	1	1	2	—	—	—	—	1	—	6
National	1	9	2	19	8	19	13	17	1	2	2	25	118

* As of March 8, 2018.

^†^ No AFP cases with paralysis onset in 2006 or 2013 had VDPV isolated from their stool sample.

^§^ Dashes indicate no cases.

**FIGURE:**
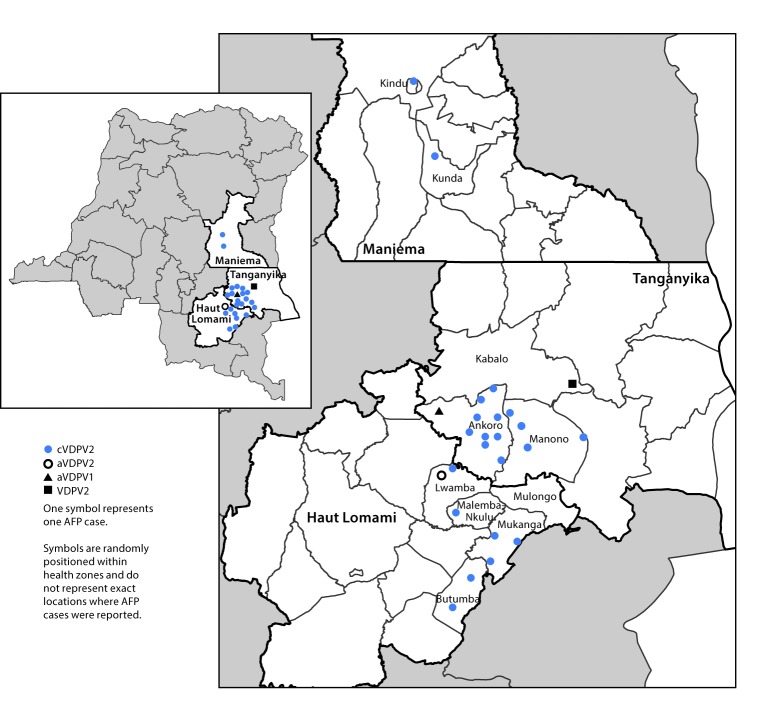
Geographic distribution of reported cases of vaccine-derived poliovirus (VDPV),* by province and health zone — Democratic Republic of the Congo, 2017^^†^^ **Abbreviations:** AFP = acute flaccid paralysis; aVDPV1/aVDPV2 = ambiguous VDPV types 1 or 2; cVDPV2 = circulating VDPV type 2; VDPV2 = VDPV type 2. * The VDPV2 case is still pending final classification. ^^†^^ As of March 8, 2018.

**TABLE 2:** Polio supplementary immunization activities (SIAs) conducted in Haut Lomami, Maniema, and Tanganyika provinces, by vaccine-derived poliovirus outbreak or event — Democratic Republic of the Congo, 2017*

Outbreak/Event	Province	Health zones with confirmed VDPV case(s)	SIA start date (oral poliovirus vaccine used), by month in 2017						
			Apr^†^ (bOPV)	Jun (mOPV2)	Jul (mOPV2)	Sep^§^ (mOPV2)	Oct^¶^ (bOPV)	Nov** (mOPV2)	Dec** (mOPV2)
Tanganyika aVDPV1/VDPV2	Tanganyika	Ankoro	Apr 9	—^††^	—	—	—	Nov 30	Dec 16
		Kabalo	Apr 9	—	—	—	—	—	—
Haut Lomami Area cVDPV2/aVDPV2	Tanganyika	Ankoro	Apr 9	—	—	—	—	Nov 30	Dec 16
		Manono	Apr 9	—	—	—	—	Nov 30	Dec 16
	Haut Lomami	Butumba	Apr 9	Jun 6	Jul 13	—	—	Nov 30	Dec 16
		Mukanga	Apr 9	Jun 6	Jul 13	Sep 14	Oct 12	Nov 30	Dec 16
		Malemba-Nkulu	Apr 9	Jun 6	Jul 13	—	Oct 12	Nov 30	Dec 16
		Lwamba	Apr 9	Jun 6	Jul 13	—	Oct 12	Nov 30	Dec 16
Maniema cVDPV2	Maniema	Kindu	Apr 9	Jun 6	Jul 20	—	—	—	—
		Kunda	Apr 9	Jun 6	Jul 20	Sep 14	—	—	—

**Abbreviations**: aVDPV1/aVDPV2 = ambiguous VDPV types 1 or 2; bOPV = bivalent oral poliovirus vaccine containing types 1 and 3; cVDPV2 = circulating VDPV type 2; VDPV2 = VDPV type 2; mOPV2 = monovalent oral poliovirus vaccine containing type 2.

*As of March 8, 2018.

^†^ The April 2017 bOPV SIA was a National Immunization Day planned prior the occurrence of these VDPV cases and was not conducted as event response.

^§^ The September 14, 2017 SIA was considered a mop-up and was only conducted in two health zones with VDPV cases.

^¶^ The October 12, 2017 bOPV SIA was conducted as part of a previously planned Local Immunization Day and was not conducted as part of outbreak response.

** The November 30, 2017 and December 16, 2017 mOPV2 SIAs were conducted in response to the cVDPV2 outbreak in the Haut Lomami area and were not related to the aVDPV1 event. The SIAs were conducted before the confirmation of the aVDPV2 in Lwamba health zone and the VDPV2 (pending classification) in Kabalo health zone.

^††^ Dashes indicate that no SIA was held during the month indicated.

## Vaccine-Derived Polioviruses

VDPVs are classified as circulating (cVDPVs) when there is evidence of community transmission; immunodeficiency-associated VDPVs (iVDPVs) when isolated from persons with primary immunodeficiency (representing a potential risk for outbreaks in areas of low poliovirus immunity*); or ambiguous (aVDPVs ) when the identity is uncertain (i.e., when investigations have not indicated ongoing transmission and the virus is not an iVDPV, including isolates identified from environmental surveillance) (*7*). VDPV types 1 or 3 are polioviruses that are >1% divergent (i.e., ≥10 nucleotide differences in the genetic sequence) from the corresponding OPV strain in the complete viral protein 1 (VP1) genomic coding region (*1*–*7*). VDPV2s are >0.6% divergent (i.e., ≥6 nucleotide differences in the genetic sequence) (*1*–*7*).

## Trivalent OPV to Bivalent OPV Switch

The 2014 World Health Assembly endorsed a strategy to reduce the risks associated with OPV polioviruses (i.e., the occurrence of vaccine-associated paralytic polio or VDPV cases) (*5*). The type 2 component of trivalent OPV (tOPV, types 1-, 2-, and 3-containing) was responsible for most cVDPV cases occurring after 2006 (*1*,*4*–*6*). Considering that WPV type 2 was declared eradicated in 2015 and in accordance with the Polio Eradication and Endgame Strategic Plan 2013–2018, all countries ceased using any type 2–containing OPV as of May 1, 2016 (*5**,**6*). A globally synchronized switch from tOPV to bivalent OPV (bOPV, type 1- and 3-containing) occurred in all OPV-using countries, including DRC (*5*,*6*). A single dose of inactivated polio vaccine (IPV) was introduced into routine immunization to mitigate the risks for an immunity gap to type 2 poliovirus (*5*).

Monovalent type 2 OPV (mOPV2) is held in a global stockpile for response to poliovirus type 2 outbreaks after the switch (*8*). The World Health Organization (WHO) Director General approves release of mOPV2 based on recommendations from the Advisory Group on mOPV2 Provision (Advisory Group) (*8*).

## Vaccine-Derived Polioviruses in Democratic Republic of the Congo

During 2004–2017 (as of March 8, 2018), 11 of DRC’s 26 provinces reported 118 cases of acute flaccid paralysis (AFP) with VDPVs isolated in stool samples (Table 1) (*1*–*6*). Until 2017, when the VDPV1 case in Tanganyika province was reported, all VDPVs had been type 2 (*1*–*6*). During 2004–2017, 63 (53%) of the 118 AFP cases with VDPV were reported in Haut Lomami province; those 63 VDPV cases were reported from eight of the province’s 16 health zones, with 34 (54%) cases from just two health zones, Kinkondja and Malemba-Nkulu (*1*–*6*).

Historically, the routine immunization program in DRC has not met global standards (*1*,*6*,*9*,*10*). Since 1996, regular preventive and outbreak response OPV SIAs have been conducted to enhance population immunity. WHO and United Nations Children’s Fund estimates of national coverage with the third dose of OPV (OPV3) in the first year of life remained <50% until 2004; coverage estimates increased to 78% by 2011 (*9*). Estimates based on vaccine doses administered and coverage survey results indicate that national OPV3 coverage has never exceeded 80% (*6*,*9*,*10*). The most recent (2013–2014) DRC Demographic and Health Survey identified subnational areas where estimated OPV3 coverage remained <60% (*10*). Introduction of IPV into the routine program in 2015 (before the tOPV to bOPV switch) has had minimal impact in building type 2 poliovirus immunity; estimated national IPV coverage was 48% in 2015 and 70% in 2016 (*9*). Thus, many areas within DRC have been and remain susceptible to the emergence of VDPVs, especially after periods of reduced numbers of OPV SIAs.

Where conducted in response to VDPVs detected before 2017, SIAs were able to interrupt transmission (*1*). No previous VDPV transmission spread nationally from the location of emergence or reappeared after apparent interruption (*1*–*5*). The 2017 cVDPV2 transmission is ongoing (*6*).

## Tanganyika aVDPV1 Event, 2017

DRC’s single case of VDPV1 was reported in April 2017 (Figure) (*6*). The patient had paralysis onset on April 1 in Tanganyika’s Ankoro health zone. The VDPV1 from this case had 25 nucleotide differences in the VP1 region from Sabin virus type 1, suggesting prolonged undetected replication. No additional VDPV1 viruses have been isolated, and the patient is not known to have an immunodeficiency; thus, to date, the case has been classified as an aVDPV1, and the occurrence is classified as a VDPV1 event per WHO’s standard operating procedures (*7*,*8*). During April 9–11, before confirmation of the case, a previously planned National Immunization Day with bOPV targeting children aged <5 years was conducted in DRC (Table 2). All health zones included in the Tanganyika clustered lot quality assurance sampling (LQAS) surveys passed the criteria for acceptable SIA performance at the 80% threshold (*8*). Clustered LQAS is a survey methodology for rapidly assessing the quality of vaccination coverage in a predefined geographic area (i.e., a “lot”); if less than nine unvaccinated children are observed in a lot of 60 children, SIA performance is said to be acceptable at the 80% threshold.^†^ No additional SIAs with a type 1-containing OPV were conducted in Tanganyika province in 2017. According to the standard operating procedures, SIAs are not required for an aVDPV1 event, although enhanced surveillance and AFP case contact investigations are recommended (*8*).

## Maniema cVDPV2 Outbreak, 2017

The first cVDPV2 patient in Maniema province had paralysis onset on March 26, 2017, in Kindu health zone (Figure) (*6*). The second case, occurring in a child residing in Kunda health zone, had paralysis onset on April 18 (*6*). No additional cases have been reported to date. Genetic analyses of the cVDPV2 viruses isolated from these cases indicated that the VP1 region sequences were identical, differing from the Sabin type 2 vaccine strain at the same 7 nucleotide positions in the VP1 region and that divergence from Sabin occurred at approximately the time of the tOPV-bOPV switch (May 2016).

The Advisory Group approved the release of mOPV2 for two SIAs targeting 276,076 children aged <5 years in eight health zones surrounding and including Kindu and Kunda in June and July (Table 2) (*6*). Results of the clustered LQAS conducted after the two SIAs indicated that Kunda health zone did not meet the criteria for acceptable SIA performance at the 80% threshold. In September, a mop-up campaign targeting 57,339 children was conducted in Kunda; clustered LQAS results indicated acceptable performance (Table 2) (*6*). After the mop-up campaign, the Advisory Group reviewed an assessment of the risk for continued viral transmission in Maniema and concluded that no additional mOPV2 SIAs were advised.

## Haut Lomami Area cVDPV2 Outbreak and aVDPV2 Event, 2017

The first patient in the cVDPV2 outbreak in the Haut Lomami outbreak area had paralysis onset on February 20, 2017 in Malemba-Nkulu health zone (Figure) (*6*). The cVDPV2 had 15 nucleotide differences from Sabin type 2 vaccine strain in the VP1 region, indicating more than 1 year of undetected circulation and therefore originating before the tOPV-bOPV switch. Six additional cVDPV2 cases with paralysis onset between March 8 and July 27 were reported in Butumba (two), Lwamba (one), and Mukanga (three) health zones. These four health zones are geographically contiguous and within Haut Lomami province (Figure) (*6*). 

The Advisory Group recommended mOPV2 for two SIAs targeting 513,820 children aged <5 years in 12 health zones (in three provinces: Haut Lomami, Lualaba, and Haut Katanga), including and surrounding the health zones where cases were reported (6). The SIAs were conducted in June and July (Table 2) (*6*). Results of clustered LQAS indicated that acceptable SIA performance in Mitwaba health zone (Haut Katanga province) was not achieved. In addition, the cVDPV2 cases in Mukanga were confirmed after the July SIA. Consequently, in September, a mop-up campaign targeting 66,006 children was conducted in Mitwaba and Mukanga with acceptable performance, based on the clustered LQAS (Table 2) (*6*). Considering the cases in Mukanga health zone with confirmation after the July SIA, the Advisory Group approved mOPV2 for two additional SIAs in the 12 health zones where the first two were conducted. Included in these SIAs were eight additional health zones (including Ankoro and Manono in Tanganyika province) contiguous with the 12 and identified as being at high risk for virus circulation because of population movement to and from the outbreak health zones, low vaccination coverage, the presence of populations that refuse vaccination, and poor AFP surveillance performance. 

Just after the Advisory Group’s approval in October 2017, the first of 13 additional, genetically linked cVDPV2 cases were confirmed in Ankoro and Manono health zones in Tanganyika province, with paralysis onset from September 14 to December 22, 2017 (Figure). In-depth genomic sequence analyses of all viral isolates from the cVDPV2 outbreak to date indicate that transmission had already extended into Tanganyika before the first outbreak response efforts were conducted in Haut Lomami province during June–September 2017; however, AFP surveillance in Tanganyika did not detect the transmission until months later. (Figure) (Table 2). The approved SIAs were conducted in December and targeted 850,002 children (Table 2). The clustered LQAS results revealing unacceptable SIA quality at the 80% threshold in numerous health zones and the paralysis onset of new cVDPV2 cases in late December 2017 indicate a need for additional SIAs in 2018.

After the December 2017 SIAs, two new VDPV2 emergences were confirmed in Haut Lomami and Tanganyika provinces. The first was in an AFP case with paralysis onset 15 November 2017 in Lwamba health zone (Haut Lomami province); this case has been classified as an aVDPV2 and the occurrence a VDPV2 event (Figure) (*8*). The second was in an AFP case in Kabalo health zone (Tanganyika province) with paralysis onset 29 December 2017; the final classification for this case is pending completion of the investigation (Figure).

## Discussion

The emergence and circulation of VDPVs during many years and over a broad geographical area is evidence of widespread suboptimal poliovirus immunity in major portions of DRC (*1*–*6*). National OPV3 coverage estimates have never exceeded 80%, and lower coverage exists in certain subnational areas (*1*,*6*,*9*,*10*). Even with preventive and outbreak response SIAs, many children remain unvaccinated or insufficiently vaccinated.

Longstanding circumstances within Haut Lomami, Maniema, Tanganyika, and other eastern provinces, including insufficient human resources, insecurity, poor roads, lack of transport and cold chain equipment, riverine and other difficult-to-reach communities, and communities historically refusing vaccination have posed challenges to routine immunization, SIA implementation, and AFP surveillance and have resulted in susceptibility to the emergence of VDPVs^§^ (*10*).

No additional cases of cVDPV2 in Maniema or VDPV1 in Tanganyika have been reported since April 2017; however, 2017 key AFP surveillance performance indicators did not meet GPEI standards in either province. The cVDPV2 transmission that spread from the administrative boundaries of Haut Lomami province to Tanganyika province had delayed detection because of surveillance gaps; thus, the initial response SIAS (June–September 2017) were of insufficient geographic scope to confine the outbreak (*6*). An external outbreak response assessment conducted in late 2017 concluded that polio immunity is inadequate to interrupt VDPV transmission in the affected areas and that AFP surveillance lacks the sensitivity to detect all remaining transmission (CDC and GPEI, unpublished data, 2017).

GPEI partners are intensifying outbreak response efforts in the Haut Lomami outbreak area and Maniema province. To achieve this, an additional surge in human and financial resources is planned. More consultants and GPEI staff will be deployed to the operational level to assist with implementation of tailored strategies to overcome the above-mentioned challenges. Planning for future SIAs will account for local circumstances, appropriate resources will be requested, and supervision will be enhanced. Intensified active AFP case search, systematic stool sample collection from AFP case contacts, and the use of telephones for “real-time” surveillance reporting will likely increase surveillance sensitivity. Environmental surveillance (i.e., wastewater collection for poliovirus testing) was established in late 2017 in Kindu (Maniema province) and in Lubumbashi (Haut Katanga province adjacent to Haut Lomami) and will continue. The risk for VDPV emergence in DRC will remain until population immunity is increased and maintained. The immediate goal is to interrupt VDPV transmission in the outbreak areas so that efforts can be turned toward improving polio vaccination and surveillance in other high risk areas in DRC.

**Summary**

**What is already known about this topic?**

Democratic Republic of the Congo has had cases of polio caused by vaccine-derived polioviruses (VDPVs) documented since 2004. The emergence of these VDPVs, which cause paralysis similar to wild polioviruses, can occur where population immunity to poliovirus is suboptimal. After an outbreak of 30 circulating VDPV type 2 (cVDPV2) cases during 2011–2012, only five VDPV2 cases were reported during 2013–2016.

**What is added by this report?**

In 2017 (as of March 8, 2018), 25 cases of VDPV were reported from three provinces, Haut Lomami, Maniema, and Tanganyika. Among the 25 VDPV cases, 22 were classified as cVDPV2, with 20 associated with an emergence that started in Haut Lomami province and spread to Tanganyika province and two associated with a separate emergence in Maniema province. Despite response efforts, transmission of these VDPVs has not yet been interrupted.

**What are the implications for public health practice?**

Risk for VDPV emergence in DRC will remain unless population immunity to poliovirus is increased and maintained. Efforts are being made as part of the current VDPV outbreak response to overcome long-standing constraints to polio vaccination. Such efforts will be extended to other regions of the country once transmission in the current outbreak areas is interrupted.
